# Underreporting of Cases in the COVID-19 Outbreak of Borriana (Spain) during Mass Gathering Events in March 2020: A Cross-Sectional Study

**DOI:** 10.3390/epidemiologia5030034

**Published:** 2024-08-09

**Authors:** Salvador Domènech-Montoliu, Maria Rosario Pac-Sa, Diego Sala-Trull, Alba Del Rio-González, Manuel Sanchéz-Urbano, Paloma Satorres-Martinez, Roser Blasco-Gari, Juan Casanova-Suarez, Maria Gil-Fortuño, Laura López-Diago, Cristina Notari-Rodríguez, Óscar Pérez-Olaso, Maria Angeles Romeu-Garcia, Raquel Ruiz-Puig, Isabel Aleixandre-Gorriz, Carmen Domènech-León, Alberto Arnedo-Pena

**Affiliations:** 1 Medical Direction University Hospital de la Plana, 12540 Vila-Real, Spain; pttcarmen@hotmail.com; 2 Public Health Center, 12003 Castelló de la Plana, Spain; charopac@gmail.com (M.R.P.-S.); aromeu96@gmail.com (M.A.R.-G.); 3 Emergency Service University Hospital de la Plana, 12540 Vila-Real, Spain; saladiego2@gmail.com (D.S.-T.); manu.msu@gmail.com (M.S.-U.); palomasatmar@gmail.com (P.S.-M.); roserblascog@gmail.com (R.B.-G.); notari_cri@gva.es (C.N.-R.); raquelruizpuig@gmail.com (R.R.-P.); 4 Health Center I and Health Center II, 12530 Borriana, Spain; delrio_alb@gva.es; 5 Nursing Service University Hospital de la Plana, 12540 Vila-Real, Spain; juancasanova83@gmail.com; 6 Microbiology Service University Hospital de la Plana, 12540 Vila-Real, Spain; gil_marfor@gva.es (M.G.-F.); perez_oscola@gva.es (Ó.P.-O.); 7 Clinical Analysis Service University Hospital de la Plana, 12540 Vila-Real, Spain; lopez_laudia@gva.es (L.L.-D.); aleixandre_isagor@gva.es (I.A.-G.); 8 Department of Medicine, University CEU Cardenal Herrera, 12006 Castelló de la Plana, Spain; carmendomenech04@gmail.com; 9 Department of Health Science, Public University Navarra, 31006 Pamplona, Spain; 10 Epidemiology and Public Health (CIBERESP), 28029 Madrid, Spain

**Keywords:** COVID-19, SARS-CoV-2, underreporting, cross-sectional, epidemiological surveillance, factors

## Abstract

Determining the number of cases of an epidemic is the first function of epidemiological surveillance. An important underreporting of cases was observed in many locations during the first wave of the COVID-19 pandemic. To estimate this underreporting in the COVID-19 outbreak of Borriana (Valencia Community, Spain) in March 2020, a cross-sectional study was performed in June 2020 querying the public health register. Logistic regression models were used. Of a total of 468 symptomatic COVID-19 cases diagnosed in the outbreak through anti-SARS-CoV-2 serology, 36 cases were reported (7.7%), resulting in an underreporting proportion of 92.3% (95% confidence interval [CI], 89.5–94.6%), with 13 unreported cases for every reported case. Only positive SARS-CoV-2 polymerase chain reaction cases were predominantly reported due to a limited testing capacity and following a national protocol. Significant factors associated with underreporting included no medical assistance for COVID-19 disease, with an adjusted odds ratio [aOR] of 10.83 (95% CI 2.49–47.11); no chronic illness, aOR = 2.81 (95% CI 1.28–6.17); middle and lower social classes, aOR = 3.12 (95% CI 1.42–6.85); younger age, aOR = 0.97 (95% CI 0.94–0.99); and a shorter duration of illness, aOR = 0.98 (95% CI 0.97–0.99). To improve the surveillance of future epidemics, new approaches are recommended.

## 1. Introduction

Determining the number of cases of an epidemic is the first and most crucial function of epidemiological surveillance. The accurate reporting of cases to the public health authorities is key to tackling an epidemic. In other words, “information is for action” [1,2]. To measure the magnitude of the COVID-19 pandemic and its evolution, typical surveillance was initially based on the reporting of cases, hospitalizations, mortality and case-fatality. However, during the first wave of the COVID-19 pandemic, a substantial underreporting of cases and deaths was observed in many regions [3,4,5,6,7]. Usually, suspected COVID-19 patients required a confirmation by a SARS-CoV-2 molecular test such as a polymerase chain reaction (PCR) to be reported; these tests were conducted in specialized laboratories. As a consequence, the case definition is very specific but less sensitive and an underestimation of the true COVID-19 incidence that took place in many countries [8,9,10].

This underreporting, considered underestimation by some authors [11], includes under-ascertainment at the community level and underreporting at the healthcare level. This relies on multiple factors such as the proportion of mild and asymptomatic cases, public health and the healthcare system, demographic characteristics, socio-economic development and political systems [12,13,14]. The underreporting of a disease can hinder the adoption of adequate prevention measures. Therefore, the consequences of underreporting can have an effect on the health of the populations and their socioeconomic situation [15,16,17].

Different methods to estimate the underreporting of COVID-19 cases and deaths have been used, including case-fatality and mortality rates, hospitalization rates, syndromic surveillance, mathematical models from reported cases, SARS-CoV-2 screening surveys, seroprevalence surveys and online open surveys [18,19,20,21,22,23,24,25]. COVID-19 seroprevalence surveys are based on the determination of anti-SARS-CoV-2 antibodies and can measure symptomatic and asymptomatic cases in large population samples [26,27,28,29].

In this context, the COVID-19 outbreak associated with the mass gathering events (MGEs) of the Falles Festival in Borriana, a municipality with approximately 35,000 inhabitants in the Valencia Community (Spain), occurred between 6 and 10 March 2020, before lockdown took place. During May and June 2020, a population-based retrospective cohort study of a representative sample of the exposed population, the Borriana COVID-19 cohort, was carried out to estimate the incidence of COVID-19 and its association with these MGEs, including a seroprevalence survey of anti-SARS-CoV-2 antibodies [30]. From January to June 2020, a total of 40 deaths from COVID-19 (mortality rate of 1.2 per 1000 inhabitants) were reported in Borriana, and 50 deaths (mortality rate of 0.29 per 1000 inhabitants) were reported in Castelló de la Plana, the capital of the province [30].

Considering that few seroprevalence surveys at the community level were conducted during the first wave of the COVID-19 pandemic and their relevance in preparation for future epidemics, the objective of this study was to estimate the reported COVID-19 cases by the health authorities in the Borriana outbreak in March 2020, and to identify factors associated with underreporting.

## 2. Materials and Methods

### 2.1. Cross-Sectional Study

A population-based cross-sectional study of this cohort was designed, and it was implemented by the Public Health Center of Castellon and the Hospital de la Plana in Vila-real, Valencia Community (Spain). Detailed information of this cohort has been described by Domènech and co-authors [30]. The study took place from January to June 2020 and 536 laboratory-confirmed COVID-19 cases were diagnosed through a seroprevalence survey with anti-SARS-CoV-2 IgM/IgG nucleocapsid antibodies. Asymptomatic cases were excluded. The laboratory technique used was a qualitative detection of antibodies against SARS-CoV-2 by an electrochemiluminescence immunoassay (ECLIA) (Elecsys^®^ Anti-SARS-CoV-2, Roche Diagnostics, Rotkreuz, Switzerland), performed at the Clinical Analysis and Microbiology Service of the Hospital de la Plana [31]. In addition, a telephone survey to obtain information about demographic characteristics, occupations, lifestyles, chronic illnesses, symptoms of COVID-19 disease, illness duration and received medical assistance for COVID-19 disease was carried out. This survey was implemented by the health staff of the Public Health Center, Emergency Service of Hospital de la Plana, and the Health Centers of Borriana, Vila-real, Onda, and La Vall d’Uixò. Lifestyles were comprehended via the following questions: Do you do habitual physical exercise? Yes/No; Do you follow a nutritional diet? Yes/No; Do you drink alcoholic beverages? Yes/No; in addition, smoking habit, weight and height were included. The telephone survey was performed from May to June 2020, and 1338 subjects took part with a participation rate of 80.5%. A more detailed description of this survey is reported by Domènech-Montoliu and co-authors [30].

To obtain the reported COVID-19 cases, the official register of notification of COVID-19 cases at the Public Health Center of Castellon was queried, including the informatics application of epidemiological surveillance analysis (AVE), considering the period January–June 2020. Following a national protocol, patients with suspected SARS-CoV-2 infection were confirmed by a positive SARS-CoV-2 PCR test or other adequate molecular test, and the confirmed cases were most of our reported cases [32,33]. PCR tests for COVID-19 cases were performed at the Microbiology Service of the Hospital de la Plana by multiple techniques and manufacturers due to the shortage of tests and material at that time.

### 2.2. Statistical Analysis

To describe the characteristics of the study population, we calculated percentages, means and standard deviations. Chi^2^ and Fisher exact tests were used for comparisons of qualitative variables, and the Kruskal–Wallis test was used for comparisons of quantitative variables.

**Figure 1:**
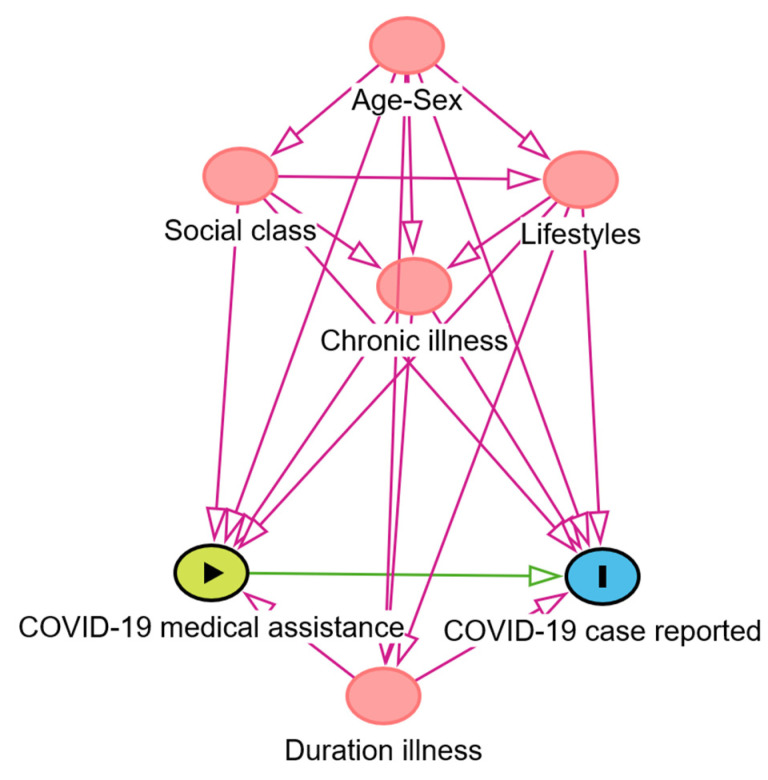
Adjusted for potential confounding factors using directed acyclic graphs (DAGs) of the COVID-19 medical assistance (exposure) effect on COVID-19 case reporting (outcome). Ancestors of exposure: age, sex, social class, lifestyles, chronic illness, duration of illness (in red) and outcome (in blue). Based on DAGitty version 3.1.

We defined underreporting COVID-19 cases as the dependent variable, and medical assistance for COVID-19 disease as a predictive variable. The independent variables were age, sex, chronic illnesses, COVID-19 illness duration in days, occupation as social class to upper class (group I higher managerial and professional occupations) versus middle and lower social classes (groups II–VI intermediate and skilled non-manual and manual occupations) [34], and lifestyles, including body mass index (kg/m^2^), smoking habit, alcohol intake, habitual physical exercise and following a nutritional diet. Logistic regression models were applied to study the associations between underreporting COVID-19 cases and the predictive and independent variables by odds ratio (OR) with a 95% confidence interval (CI). To control potential confounding factors, a study of the medical literature was addressed, and directed acyclic graphics (DAGs) analysis were employed [35,36] with the DAGitty program version 3.1 [37]. Figure 1 describes the relationships between the exposure (COVID-19 medical assistance), the ancestors of exposure and the outcome (COVID-19 case reported). Statistical program Stata^®^ 14 version 2 was used for all the calculations.

The study had the approval of the director of the Public Health Center of Castellon and the management of the Health Department of La Plana. This study was exempt from the Ethics Review Board approvals protocol following the Spanish legislation as part of the public health surveillance of the COVID-19 pandemic.

## 3. Results

**Figure 2:**
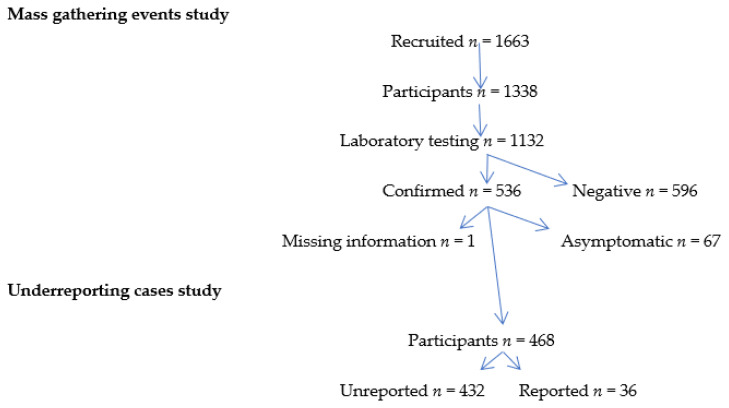
Flow diagram showing the population under study in the underreporting of COVID-19 cases in Borriana during the period January–June 2020.

Of a total of 536 laboratory-confirmed symptomatic COVID-19 cases in the outbreak, 67 asymptomatic cases were excluded, and 1 case showed missing information (Figure 2). A total of 468 cases were thus finally included in the study (99.8%). Among these, only 36 (7.7%) cases were reported as COVID-19 cases by the Public Health Center, resulting in an underreporting proportion of 92.3% (95% CI 89.5–94.6%), or 13 unreported cases for every reported case. Cases reported by the Public Health Center included 34 (94.4%) with a positive PCR and 2 (5.6%) with positive anti-SARS-CoV-2 antibodies. All the unreported cases were laboratory-confirmed by anti-SARS-CoV-2 antibody serology.

The symptoms of reported and unreported COVID-19 cases are shown in Table 1. Reported cases presented higher clinical severity with significant differences in symptoms such as fever, cough, sore throat, diarrhea, dyspnea and pneumonia. Twelve reported cases required hospitalization, and one death attributable to COVID-19 took place during the study period.

Characteristics of reported and unreported COVID-19 cases are shown in Table 2. Unreported cases were significantly younger than reported cases. Unreported cases received significantly less medical assistance for COVID-19 disease and had a shorter duration of illness than reported cases. The lack of a chronic illness was higher in the unreported cases. The middle and lower social classes were significantly predominant in the unreported cases. Obesity, current smoking, alcohol intake, habitual physical exercise and following a nutritional diet were not associated with the underreporting group.

Crude and adjusted logistic regression analyses of factors associated with underreporting are shown in Table 3. Significant factors associated with underreporting were a younger age, lack of medical assistance for COVID-19 disease, shorter duration of illness and absence of a chronic illness. The middle and lower social classes were significantly more related to underreporting compared with the upper class. The surveyed lifestyle factors were not significantly associated with underreporting.

## 4. Discussion

Our results suggest that the underreporting of symptomatic COVID-19 cases in the Borriana COVID-19 cohort was very high during the first wave of the COVID-19 pandemic. Factors associated with this underreporting of cases were young age, no received medical assistance for COVID-19 disease, short illness duration, absence of a chronic illness and belonging to the middle and lower social classes.

This COVID-19 outbreak took place during mass gathering events with a massive exposition of SARS-CoV-2 with a 39.2% attack rate [30]. This exposition occurred between 6 and 10 March 2020, five days before the Spanish lockdown was enforced. Cases had a milder illness and were not reported in line with the Spanish official publication of the first-wave COVID-19 pandemic [33]. This type of COVID-19 outbreak may illustrate how the COVID-19 epidemic spread in some regions, as well as how mass gathering events during February and early March 2020 could have played an important role in the spread of COVID-19 cases around Spain and other countries [38,39,40,41,42,43].

Estimations of underreporting of cases and deaths showed important geographic variation. However, this underreporting was very elevated during the first wave of the COVID-19 pandemic in most countries [4,44,45,46,47]. In seroprevalence studies, different methodologies and analytic techniques have been employed to estimate the dimensions of the COVID-19 pandemic and the proportion of asymptomatic cases [29,48]. Considering some population-based seroprevalence surveys detecting anti-SARS-CoV-2 antibodies, the number of unreported cases for every reported COVID-19 case presented considerable differences, from 2.8 cases in Santiago de Chile [49] to 25.5 cases in Eswatini, Southern Africa [26,27,50,51,52,53]. Our results are consistent with the study of Sierra and co-authors [54], where the sensitivity of the Spanish surveillance system was 9.7% (95% CI 8.96–10.29) with 13 unreported cases for every reported case following the national seroprevalence survey of Pollan and co-authors [28].

With respect to Spain, we could address some causes for the low reporting, considering an unprecedented situation with a new disease and the countrywide lockdown. The causes could include the high proportion of milder and asymptomatic infections, predominant reported COVID-19 cases with positivity under SARS-CoV-2 PCR, an insufficient follow-up of infected cases and contacts, restrictions and barriers for medical care access, limited capacity of SARS-CoV-2 laboratory testing, prioritization of medical assistance and laboratory SARS-CoV-2 PCR testing for patients with severe illness, restriction in population mobility and social isolation in the context of subordinate public health [4,14,33,55]. This underreporting could be responsible for the delay of the health authorities to carry out mitigation strategies [56]. An official document about the epidemic [57] indicated that the Spanish system of health was not sufficiently prepared for the COVID-19 pandemic, lacking the stocks of material necessary to tackle a respiratory virus pandemic, having weak information systems and insufficient diagnostic recourses. In addition, the healthcare system was overwhelmed and critical services were saturated during this first wave [33].

Factors associated with underreporting such as no medical assistance for COVID-19 disease, young age, absence of a chronic illness and a short illness duration suggest a mild illness and few severe cases, which aligns with other studies [50,53,58]. Patients belonging to the middle and lower social classes were more underreported than upper class patients, suggesting better access to healthcare in the latter. Less reporting of COVID-19 cases in manual occupations such as waiter or taxi-drivers was observed in Norway [59]. In our study, the surveyed lifestyle factors were not associated with underreporting, but in other studies, obesity has been associated with a higher possibility to be tested and to be COVID-19-positive [60].

Our study presents some strengths and limitations. As strengths, we present a representative sample of the population exposed to SARS-CoV-2 with a high participation. In our study, controlling for potential confounding factors was carried out by logistic regression models, and the sensitivity and specificity of the technique for anti-SARS-CoV-2 antibodies were elevated. Asymptomatic COVID-19 cases were excluded in the estimation. Our proportion of asymptomatic cases was low, 12%, compared with reviews and meta-analyses [61]. As limitations, we include that underreporting was studied during a COVID-19 outbreak, and considering the elapsed time between the exposition and the start of the study, some recall and misclassification biases could have occurred. It is worth considering that anti-SARS-CoV-2 antibodies decline over time, which could impact the identification of cases after the initial disease onset. Yet, our study was implemented three months after the mass gathering events, and 99% of anti-SARS-CoV-2 antibody persistence was found in a study of this cohort in October 2020 [62]. Finally, the cross-sectional design of our study can only establish associations of potential risk factors and no cause–effect relationships.

In the public health arena, many voices have indicated the need for a change in order to improve the surveillance of infectious diseases [54,63,64,65,66], and a critical review of the surveillance methods was performed in England [67]. Considering that the possibility of future epidemics is not remote, novel approaches to surveillance are recommended. Five areas could be contemplated. First, a community approach with continued studies of representative population samples and household surveys, including studies of the incidence of infectious diseases and serological surveys, attendance to emergency departments, hospitalizations, visits to primary healthcare centers, syndromic surveillance and the determination of high-risk groups [67,68,69]. Second, digital surveillance, which experienced rapid development during the COVID-19 pandemic, has been suggested to be useful for tracking COVID-19 cases [70]. This include online self-reported population surveys or digital apps [71,72], mobile apps with different uses such as risk assessment and contact tracing [73,74] and big data and infodemiology used to obtain useful public health information [75]. Third, surveillance can be improved through mathematical models to estimate the true dimensions of epidemics, including the asymptomatic infections with the application of artificial intelligence, which could be useful to complement more traditional epidemiological methods [76,77]. Some practical approaches are being implemented such as a new surveillance index [78] and a calculated refined reproduction number [79]. Fourth, wastewater surveillance determining the presence of SARS-CoV-2 in the sewage system has undergone considerable development in epidemic detection [80,81]. Finally, an area of particular interest is genomic surveillance that allows the detection of new viral variants to make valuable use against SARS-CoV-2 in household transmission, outbreak detection and national variant surveillance [82,83,84,85,86]. In addition, the cycle threshold of SARS-CoV-2 RNA PCR results could be useful to forecast COVID-19 epidemics [87]. From the results of our study, some specific measures could be recommended to improve the reporting, including a more sensitive case definition, considering clinical symptoms and exposures, using serological tests of SARS-CoV-2, increasing medical assistance in epidemic situations with better access for the middle and lower social classes, and extending medical assistance to less severe cases.

## 5. Conclusions

During the first wave of the COVID-19 pandemic, the underreporting of COVID-19 cases was very high, indicating insufficient preparedness for large epidemics. Our study pinpoints variables associated with underreporting, opening new avenues for improving it being addressed. To improve the surveillance of future epidemics, new approaches are recommended.

## Figures and Tables

**Table 1:** Symptoms of reported and unreported COVID-19 cases in the Borriana COVID-19 cohort, during January–June 2020.

Symptoms	Reported Cases N = 36 (%)	Unreported Cases N = 432 (%)	*p*-Value
Weakness	25 (69.4)	240 (56.6)	0.118
Fever	29 (80.6)	221 (51.2)	0.001
Loss of smell and/or taste	22 (61.1)	238 (55.2) ^1^	0.601
Cough	24 (66.7)	202 (46.9) ^2^	0.024
Sore throat	19 (52.9)	138 (31.9)	0.016
Coryza	12 (33.3)	135 (31.3)	0.852
Headache	24 (66.7)	193 (44.9) ^3^	0.014
Myalgia	23 (64.9)	218 (50.5)	0.164
Dermatologic lesions	7 (19.4)	46 (11.1) ^4^	0.171
Diarrhea	17 (47.2)	110 (25.5)	0.010
Vomiting	4 (11.1)	25 (5.8)	0.266
Dyspnea	15 (41.7)	7 (1.6)	0.000
Pneumonia	11 (30.6)	0 (0.0)	0.000

^1^ Missing answer from 1 participant. ^2^ Missing answer from 1 participant. ^3^ Missing answer from 2 participant. ^4^ Missing answer from 18 participants.

**Table 2:** Characteristics of reported and unreported COVID-19 cases in the Borriana COVID-19 cohort. January–June 2020.

Variables	Reported Cases N = 36 (%)	Unreported Cases N = 432 (%)	*p*-Value
Age (years) ± SD ^1^	45.7 ± 16.4	37.2 ± 16.5	0.001
Male	14 (38.8)	162 (37.5)	0.860
No medical assistance for COVID-19 disease	2 (5.6)	236 (54.6)	0.000
Duration illness (days) ± SD ^2^	24.2 ± 21.9	10.5 ± 14.9	0.000
No chronic illness ^3^	14 (38.8)	295 (68.3)	0.001
Middle and lower social classes ^4,5^	25 (69.4)	375 (87.4)	0.009
Body mass index (kg/m^2^) ± SD ^6^	26.4 ± 5.8	25.0 ± 5.0	0.183
Obesity ≥ 30 kg/m^2^ ± SD ^7^	10 (27.8)	69 (16.1)	0.102
Current smoker ^8^	3 (8.3)	64 (15.3)	0.333
Alcohol intake, yes ^9^	11 (30.6)	100 (23.8)	0.417
Habitual physical exercise, yes	17 (47.2)	256 (59.3)	0.164
Nutritional diet, yes ^10^	10 (27.8)	75 (17.4)	0.121

^1^ Standard deviation. ^2^ Missing answer from 48 participants. ^3^ Missing answer from 5 participants. ^4^ Missing answer from 4 participants. ^5^ Higher managerial and professional occupations (group I). ^6^ Missing answer from 3 participants. ^7^ Missing answer from 4 participants. ^8^ Missing answer from 13 participants. ^9^ Missing answer from 11 participants. ^10^ Missing answer from 1 participant.

**Table 3:** Factors associated with underreporting COVID-19 cases in the Borriana COVID-19 cohort by logistic regression. Crude and adjusted odds ratio (OR) and (aOR); 95% confidence interval (CI).

Variable	OR	95% CI	aOR	95% CI	*p*-Value
Age (years) ^1^	0.97	0.95–0.99	0.97	0.94–0.99	0.003
Male ^2^	0.94	0.47–1.89	1.16	0.94–2.35	0.690
No medical assistance for COVID-19 disease ^3^	17.6	4.86–86.30	10.83	2.49–47.11	0.001
Duration illness (days) ^4^	0.98	0.96–0.99	0.98	0.97–0.99	0.037
No chronic illness ^5^	3.34	1.66–6.72	2.81	1.28–6.17	0.010
Middle and lower social classes ^6^	3.06	1.42–6.56	3.12	1.42–6.85	0.005
Body mass index (kg/m^2^) ^7^	0.94	0.88–1.01	0.98	0.91–1.06	0.651
Obesity ≥ 30 kg/m ^2,7^	0.50	0.33–1.08	0.64	0.28–1.47	0.294
Current smoker ^8^	1.98	0.59–6.67	2.14	0.62–7.41	0.228
Alcohol intake, yes ^9^	0.70	0.34–1.49	0.59	0.26–1.33	0.203
Habitual physical exercise, yes ^10^	1.63	0.2–3.21	1.91	0.91–4.00	0.085
Nutritional diet, yes ^11^	0.55	0.25–1.18	0.65	0.29–1.48	0.307

^1^ Adjusted for sex. ^2^ Adjusted for age. ^3^ Adjusted for age, sex, social class, chronic illness, illness duration. ^4^ Adjusted for age, sex, chronic illness, smoking, alcohol intake, physical exercise, nutritional diet, body mass index. ^5^ Adjusted for age, sex, social class, smoking, alcohol intake, physical exercise, nutritional diet, body mass index. ^6^ Adjusted for age, sex. ^7^ Adjusted for age, sex, social class, smoking, alcohol intake, physical exercise, nutritional diet. ^8^ Adjusted for age, sex, social class, alcohol intake, physical exercise, nutritional diet, body mass index. ^9^ Adjusted for age, sex, social class, smoking, physical exercise, nutritional diet, body mass index. ^10^ Adjusted for age, sex, social class, smoking, alcohol intake, nutritional diet, body mass index. ^11^ Adjusted for age, sex, social class, smoking, alcohol intake, physical exercise, body mass index.

## Data Availability

The data of the study can be consulted if the authors are requested.
